# Organoboronic acids/esters as effective drug and prodrug candidates in cancer treatments: challenge and hope

**DOI:** 10.1080/14756366.2023.2220084

**Published:** 2023-06-15

**Authors:** Mothana K. Al-Omari, Mai Elaarag, Raed M. Al-Zoubi, Ahmad R. Al-Qudimat, Ayman A. Zarour, Enas A. Al-Hurani, Zainab E. Fares, Leena M. Alkharraz, Mohanad Shkoor, Abdulilah D. Bani-Yaseen, Omar M. Aboumarzouk, Aksam Yassin, Abdulla A. Al-Ansari

**Affiliations:** a Department of Chemistry, Jordan University of Science and Technology, Irbid, Jordan; b Surgical Research Section, Department of Surgery, Hamad Medical Corporation, Doha, Qatar; c Department of Biomedical Sciences, QU-Health, College of Health Sciences, Qatar University, Doha, Qatar; d Faculty of Medicine, Univerity of Debrecen, Debrecen, Hungary; e Department of Chemistry, Université de Montréal, Montréal, Canada; f Department of Chemistry and Earth Sciences, Qatar University, Doha, Qatar; g College of Medicine, Qatar University, Doha, Qatar; h School of Medicine, Dentistry and Nursing, The University of Glasgow, Glasgow, UK; i Center of Medicine and Health Sciences, Dresden International University, Dresden, Germany

**Keywords:** Boronic acid, cancer disease, enzyme inhibitor, mechanism, drug

## Abstract

Boronic acids/esters have recently emerged in the field of medicinal and pharmaceutical research due to their exceptional oxophilicity, low toxicity, and unique structure. They are known as potent enzyme inhibitors, cancer therapy capture agents, and can mimic certain types of antibodies to fight infections. They have been designed and developed into drugs, and this approach has emerged in the last 20 years. Five boronic acid drugs have been approved by the FDA and Health Canada, two of which are used to treat cancer, specifically multiple myeloma. The purpose of this review is to investigate boronic acid/ester derivatives as potential pharmaceutical agents as well as the mechanism of action. It will concentrate on six types of cancer: multiple myeloma, prostate cancer, breast cancer, lung cancer, cervical cancer, and colon cancer. Some newly developed boron-containing compounds have already demonstrated highly promising activities, but further investigation is required before final conclusions can be drawn.

## Graphical Abstract

**Figure:**
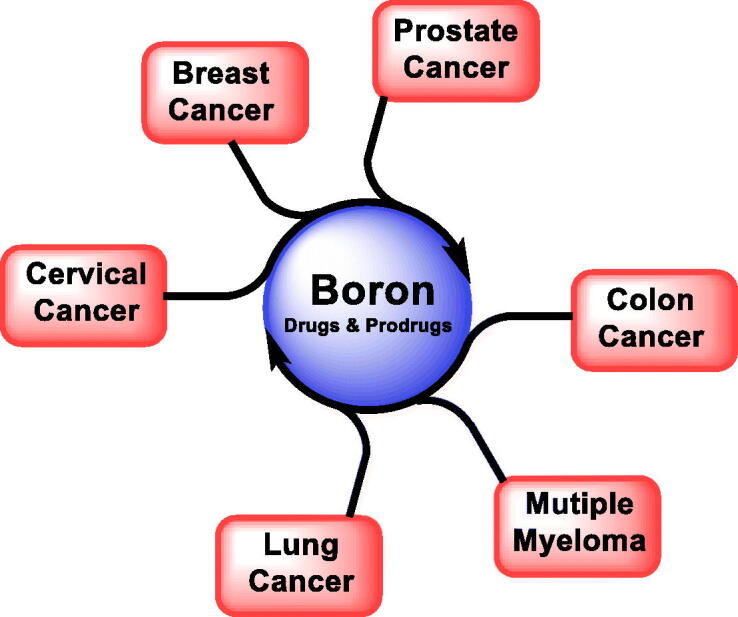

## Introduction

In the past, several pharmaceutical industries have started to develop organic molecules derived from nature. It can be argued that the creation of drug discovery with its rare, presented ingredients is a daring endeavour^1^. Enzyme inhibition plays a critical role in drug discovery since many diseases and disorders result from the abnormal activity of specific enzymes. Enzymes are biological catalysts that catalyse various biochemical reactions in living organisms. Inhibiting the activity of specific enzymes can help to regulate or block the biological process that leads to the disease^2^.

Enzyme inhibition can be achieved through different mechanisms, including uncompetitive inhibition, non-competitive inhibition, and competitive inhibition. Competitive inhibition involves a molecule that competes with the substrate for the enzyme’s active site^3^. Non-competitive inhibition involves a molecule that binds to a location on the enzyme other than the active site, thereby altering the enzyme’s conformation and reducing its activity. Uncompetitive inhibition involves a molecule that binds to the enzyme-substrate complex, preventing the enzyme from catalysing the reaction^4^.

In drug discovery, enzyme inhibitors are often used as therapeutic agents to treat various diseases. For example, enzyme inhibitors can be used to treat viral infections, cancer, and metabolic disorders. For example, inhibitors of HIV protease are used to treat HIV infections, while inhibitors of histone deacetylases (HDACs) are used to treat cancer^5^^,^^6^. Enzyme inhibitors can also be used to improve the efficacy of existing drugs by slowing down their metabolism or enhancing their activity.

Drug discovery strategies are based on medical needs that are addressed through various drug development techniques, which can be difficult and require conventional thinking to exploit the special unique properties of these components, one of which is boronic acid/ester. It is an important chemical entity for humans, animals, and plants^7^. It is essential for bone and brain health, increases the level of antioxidant enzymes, raises magnesium absorption, beneficial for sex hormones, and vitamin D. In addition, it has been shown to lower biomarker levels that cause inflammation, such as tumour necrosis factor-alpha (TNF-α) and highly sensitive C-reactive protein (hs-CRP). Also, hs-CRP and TNF-α decrease immediately as boron levels increase in the human body. The hs-CRP and TNF-α are responsible factors for some types of cancer^8^^,^^9^. It has also led to great preventive and therapeutic effects in many cancer types, such as multiple myeloma (MM), prostate, breast, lung, cervical, and colon cancers^9^. Boronic acid/ester has been successfully incorporated into cancer treatments and therapy mainly due to its remarkable oxophilicity and low toxicity levels in the body as small derivatives tend to be water soluble and are often excreted through the kidneys unmetabolized^10^. Oxophilicity increases binding which converts the geometry from a trigonal form to a tetrahedral form that facilitates the movement of receptors through the cell wall^11^.

Organoboron’s main strength as an anticancer agent is its ability of proteasome inhibition, defined as a sophisticated protease found to achieve hydrolysis of client proteins in an efficient and selective pathway^6^. Additionally, it is polymerised to create a biomarker for regulated proteolysis in eukaryotic cells by collaborating with ubiquitin^12^. Scorei et al. concluded that organoboron is not only an anticancer agent but also has the ability to prevent cancer in the first place^13^^,^^14^.

**Figure 1.**
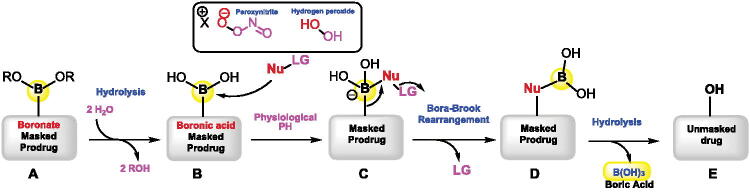
The release mechanism of active prodrug from boron-masking using direct C–B bond oxidation/rearrangement strategy.

Boronic acids/esters have been widely developed and used in drug design and discovery for the protection and masking of groups that serve as a drug reservoir and can trigger tumour microenvironmental abnormalities such as high levels of reactive oxygen species (ROS) and overexpressed enzymes^15–18^. Boronate esters A (Figure 1) are usually hydrolysed under aqueous biological conditions, giving free boronic acid B and alcohol derivatives^19^. The carbon-boron bond in boronic acids/esters can be further oxidised using reactive species such as hydrogen peroxide, peroxynitrite and hypochlorite anion^20^. This remarkable oxophilicity of boron with an empty p orbital makes it available for nucleophilic attack by the oxygen of the reactive species, forming an activated tetrahedral boronate intermediate C^21^. The Bora-Brook rearrangement involves the migration of carbon to oxygen giving a borate derivative D, which is then hydrolysed to give the desired alcohol drug E and boric acid^22^. It is worth noting that under physiological pH, the oxidation/Bora-Brook rearrangement step using peroxynitrite is much faster (106 times) than the conversion using hydrogen peroxide. This is more likely due to the neutral form of hydrogen peroxide (H_2_O_2_) at physiological pH compared to the anionic form of peroxynitrite^20^.

The boronic acid/ester functionalities can reversibly react with aromatic or aliphatic alcohols^17^^,^^18^^,^^20^^,^^23^, enols^24^ or cis-diol systems commonly found in glycoproteins and sugar molecules^25^. In addition, boronic acid/ester-containing prodrugs of aliphatic or aromatic hydroxylated moieties in drug scaffolds have also been designed to increase bioavailability by reducing first-pass metabolism^26^.

This type of masking is commonly usually used as temporary moieties and can be easily removed under physiological conditions. It can be attached either directly to the core structure of the prodrug or through a spacer such as a carbonate or a carbamate using amino or hydroxyl functionality^27^. The length and volume of the spacer should contribute to better masking without affecting the biological activity, which can be removed by oxidation, and a subsequent rearrangement process resulted in the free release of the parent prodrug at physiological pH.

**Figure 2.**
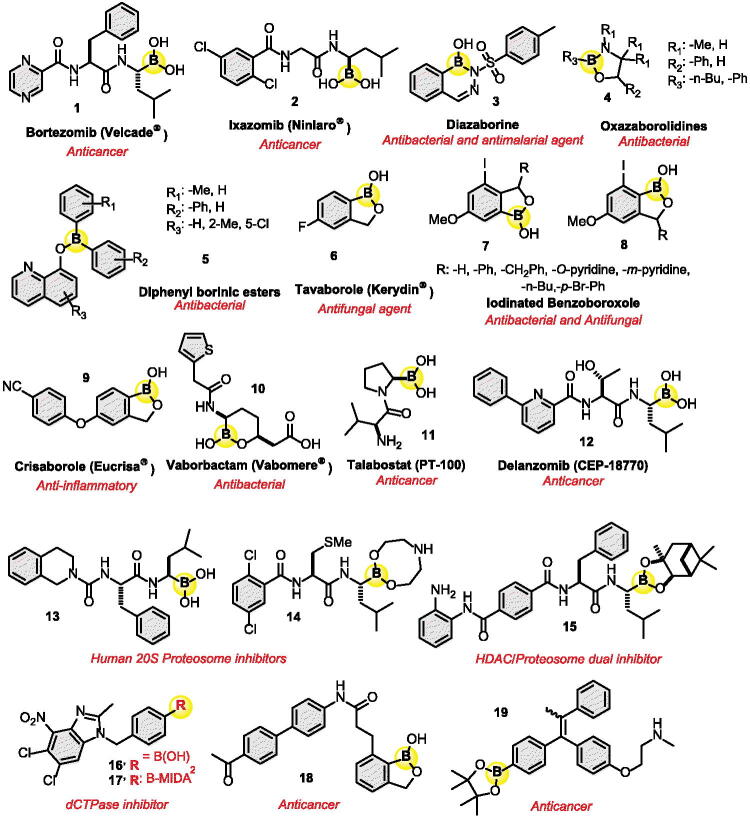
Some biological active organoboron derivatives in medicine.

There are various biomedical uses for organoboron medications as shown in Figure 2^28^. It is used as a boronic acid derivative in medications to treat MM, such as bortezomib **1** (Velcade®) and ixazomib **2** (Ninlaro®) and in boron neutron capture therapy (BNCT) for other cancer treatments. The use of boron as an entity in the synthesis of bioorganic derivatives is well known^29^.

Organoboron derivatives can also be used as antibacterial agents^30^ or antifungal agents such as diazaborine **3**^31^, oxazaborolidine **4**^32^, diphenyl borinic ester **5**^33^, tavaborole **6** (Kerydin®)^34^, and iodinated benzoboroxoles **7**,**8**^35^. In addition, crisaborole **9** (Eucrisa®)^36^ is used for the treatment of mild-to-moderate atopic dermatitis (eczema) in adults and children. Another organoboron medication called vaborbactam **10** (Vabomere®) is used for urinary tract infections and pyelonephritis^37^.

Herein, this article discusses the challenges and opportunities associated with using boron-containing drugs and prodrugs in enzyme inhibition-based drug discovery in six types of cancer: multiple myeloma, prostate cancer, breast cancer, lung cancer, cervical cancer, and colon cancer. These cancers were chosen because they are the most discussed cancers and demonstrated excellent results in the literature where boron derivative has been used in its clinical treatment or pre-clinical whether in vivo or *in vitro*.

## Approved boronic acid anticancer drugs

Cancer is estimated as the cause of one-sixth of all deaths worldwide and is the second leading cause of death with almost 9.6 million cancer deaths in 2018^38^. To this day, drug therapy and surgery are the first methods of choice in cancer treatment^39^, and in this perspective, cancer drug medications typically cause unwanted side effects, toxicity and drug resistance. Therefore, more research on improved cancer medications is still needed to encounter these challenges.

Bortezomib **1**: marketed under the name Velcade®, is a boron-containing medication and is the first FDA-approved proteasome inhibitor used for the treatment of MM. It got approved by the FDA in 2003^40^, and by Health Canada in 2008^41^. The structure was originally discovered through peptide aldehydes that are found to bond covalently to nucleophilic threonine residue through co-crystallization. However, boronic acid analogs were tested and showed high potency since aldehydes are not suitable for further drug development^42^. Its mechanism of action includes the blocking of the ubiquitin-proteasome pathway attaching to the chymotrypsin-like (CT-L) site of the 20S proteolytic core of the 26S proteasome^43^. More specifically, it binds to the catalytic site of the proteasome, preventing it from cleaving proteins. This leads to the accumulation of misfolded or damaged proteins within the cancer cell, which triggers a series of cellular events culminating in apoptosis^43^^,^^44^. This is important in myeloma cells because it involves inhibiting the proteasome complex and disrupting various cellular processes, ultimately leading to cancer cell death.

Bortezomib is the standard treatment for multiple myeloma and the first line of therapy recommended for patients with MM. For adults with previously untreated MM, bortezomib is given as a weekly injection in combination with Prednisone (a glucocorticoid medication) and Melphalan (a MM medication) for nine cycles. Every IV injection contains 1.3 mg/m^2^ of bortezomib. It is given twice a week in cycles 1–4 and given once a week in cycles 5–9. For adults with relapsed MM, the 1.3 mg/m^2^ IV dose is given twice a week for two weeks with a 10-day rest period or once a week for four weeks followed by a 13-day rest period^45^.

Ixazomib **2**: is another boronic acid medication, marketed under the name Ninlaro®. It is the 2nd proteasome inhibitor boron-containing drug to get approved by the FDA in 2015^46^ and by Health Canada in 2016^47^ to also treat MM. Like bortezomib 1, the inhibition of proteasome activity by ixazomib 2 leads to the accumulation of misfolded or damaged proteins within the cancer cell, resulting in cellular stress and ultimately leading to apoptosis. Due to its unique structure, it rapidly absorbed into the bloodstream and distributed throughout the body.

Ixazomib **2** has found to be even more potent and less likely to cause side effects. It does not only target CT-L, but also trypsin-like (T-L) and caspase-like (C-L) subunits^44^. Therefore, it is reported to have a higher selectivity and is usually given to patients who develop resistance to bortezomib **1**^48^. Despite the fact that bortezomib **1** and ixazomib **2** are both reversible inhibitors of the same class with comparable potencies, ixazomib **2** has a substantially a shorter protease dissociation half-life for proteasome binding than bortezomib **1** (18 vs. 110 min *t*_1/2_),^49^^,^^50^ a greater ability to permeate tissues, higher enrichment in tumour tissue, and the ability to trigger apoptosis of drug-resistant cell lines, resulting in a better anti-MM action than bortezomib.^51^ Bortezomib’s slow dissociation rate from the proteasome in red blood cells limits medication distribution. Therefore, ixazomib **2** had been developed to address this problem. Ixazomib’s fast off-rate allows it to associate and disassociate with various proteasomes, allowing it to reach more cells and spread more widely for achieving a therapeutic effect while minimising toxicity^49^.

In addition, bortezomib **1** is an injectable medication while ixazomib **2** is an oral medication. It has three different doses; 2.3 mg, 3 mg, and 4 mg. It is given to patients who have had at least one previous therapy and is used in combination with dexamethasone (a glucocorticoid medication) and lenalidomide (a MM medication). For adults, 4 mg is given through a 28-day cycle on days 1, 5, and 18 with a two-week resting period until the next cycle begins. The doses may be adjusted^52^. Ixazomib is currently being tested as combination therapy with other drugs to treat MM, as many patients may require multiple lines of treatment^48^^,^^52^^,^^53^.

## The safety profile of approved B-containing anticancer drugs

Generally, safety assessment is a vital process in drug discovery and development that makes decisions about the drug candidates to be approved by FDA as available drugs in the market. This process does not end at this stage but continues through post-marketing safety surveillance, which sometimes resulted the drug being withdrawn from the market.

A comprehensive drug assessment safety profile is built by taking the drug candidate through several phases; starting from pre-clinical trials, which include *in vitro* and in vivo testings and crossing up the clinical trial studies (Phase I, II, III) until approved by the FDA to get into the market and continue to phase IV (the post-marketing monitoring)^54^. These lengthy processes take a long time, estimated to take 12–15 years^55^. The two major tests that cause the drugs to be withdrawn are hepatotoxicity and cardiotoxicity^56^. Bortezomib **1** (Velcade®) and ixazomib **2** (Ninlaro®) can be administered with manageable side effects, with the peripheral nervous system (PNS), circulatory system (hematological toxicity) and the gastrointestinal (GI) system to be the most affected (Table 1)^57^.

**Table 1.** The safety profile of Velcade® and Ninlaro®.

Toxicity/side effect	Bortezomib (VELCADE®)	Ixazomib (NINLARO®)	Managing the side effect	Reference
Peripheral neuropathy	Grade ≥ 2, 24–39% Grade ≥ 3, 6–15%)	Grade 1 (18%) Grade 2 (11%) Grade 3 (2%)	Monitor the symptoms, decrease the dose or decrease the dose-intense schedule	^58^
Thrombocytopenia	Grade 3 (35%) Grade 4 (43%) Severe (30%)	Grade 3 (13%) Grade 4 (17%)	Monitor blood count regularly and adjust dosing accordingly	^59^
Cardiac toxicity	≤1%	No significant cardiovascular risk	Monitor patients with existing heart diseases	^60^
Gastrointestinal toxicity (diarrhoea, nausea, and vomiting)	Diarrhoea (55–58%, severe 7–8%) Nausea (57–64%, severe 2–7%) Vomiting (35–36%, severe 3–7%)	All grades: Diarrhoea (52%) Nausea (32%) Vomiting (26%) Severe (2–3%)	Use of antiemetic and antidiarrheal medications or fluid replacement	^57–59^
Hepatotoxicity	≤1%	≤1%	Monitor hepatic enzymes	^60^

The incidence rate of some side effects of bortezomib among patients is influenced by the route of administration; subcutaneously (SC) vs intravenously (IV)^58^. In the case of MM, peripheral neuropathy has been reported in phase 3, it occurring with toxicity grade 2 in 24% of patients administered bortezomib SC vs 39% in the IV administration group, while the toxicity percentage for grade 3 was 6% for SC vs 15% for IV^58^. The incidence rate of peripheral neuropathy for ixazomib **2** administered orally was 18% of patients for grade 1, 18% for grade 2, and 2% for grade 3^58^. The incidence rate of thrombocytopenia that occurred in patients treated with bortezomib showed significant rates in grades 3 and 4, in this cases reaching the severity which was 35%, 43%, and 30% of patients, respectively^59^. Whereas in groups treated with ixazomib, the rates were 13% for grade 3 and 17% for grade 4^59^. Gastrointestinal toxicity manifests in several common symptoms, such as diarrhoea, nausea and vomiting. All grades showed a high incidence rate for both bortezomib and ixazomib^58^^,^^59^. Finally, cardiotoxicity and hepatotoxicity are rare with both drugs^60^.

While both bortezomib and ixazomib are medications used to treat multiple myeloma, they differ in their side effects^61^. Both medications may cause gastrointestinal symptoms such as nausea, vomiting, diarrhoea, and constipation, as well as peripheral neuropathy and fatigue. However, bortezomib has a higher incidence and severity of peripheral neuropathy than ixazomib. In contrast, ixazomib may cause liver toxicity, blood clots, and cardiac effects such as heart failure or changes in heart rhythm, which are rare side effects not typically associated with bortezomib. Additionally, bortezomib may cause more severe blood count abnormalities than ixazomib, including low platelets and anaemia. Skin reactions, such as rash or itching, are common with both medications but may require topical treatment for ixazomib-induced reactions.

## Boron-containing anticancer drugs that failed clinical trials

The successful development of bortezomib and ixazomib led to the discovery of other potential boronic acid drugs^62^. Talabostat (PT-100) **11**, also called Val-Boro-Pro, has been discovered for the treatment of non-Hodgkin’s lymphoma, leukaemia, lung cancer, pancreatic cancer and melanoma^63^. It is dipeptidyl peptidase (DPP) and fibroblast activation protein-alpha (FAP) inhibitors that cause cell death through the upregulation of cytokines and chemokines that boosts the immune system response^63^. The DPP cleaves Xaa-Pro (an amino acid that’s adjacent to proline), and was designed by replacing Xaa-Pro with boronic acid instead of the carboxylic acid to be able to bind to catalytic serine^64^. However, it has failed phase III clinical trials as its results have not been conclusive and its mechanism of action is not fully known^62^. The most commonly reported side effects of talabostat included gastrointestinal disturbances such as nausea, vomiting, and diarrhoea. Other potential side effects may include fatigue, headache, and injection site reactions^65^.

Research into proteasome inhibitors continues also due to the resistance that bortezomib causes in some patients^66^. Delanzomib (CEP-18770) **12** has been tested in clinical trials for the treatment of solid tumours, MM, and non-Hodgkins lymphoma. Delanzomib works like bortezomib **1** and ixazomib **2** by inhibiting the proteasome’s actions and stopping the nuclear factor kappa B alpha (NF-KB α) which results in apoptotic tumour cell death. It passed the non-Hodgkins and solid tumour test in the phase I trials but failed the MM test in phase II trials^67^. Some of the most commonly reported side effects of delanzomib included gastrointestinal disturbances such as nausea, vomiting, and diarrhoea. Other potential side effects may include fatigue, headache, and fever^68^. In preclinical studies, delanzomib has been shown to have some toxicity to the liver and kidneys.

## Other anticancer boron-containing drugs discovery

Han et al. did a structure-activity relationship (SAR) approach between different proteasome inhibitors and discovered boronic acid **13** (Figure 2). It showed activity against the 20S proteasome and activity against 11 cancer cell lines during *in vitro* assays. In vivo mice assays showed anti-tumour activity and stronger pharmacokinetics than bortezomib. This compound is currently under pre-clinical trials in response to these promising results^69^. Moreover, Lei et al. investigated a proteasome inhibitor for the treatment of MM in addition to triple-negative breast cancer (TNBC) by a SAR test combining both bortezomib and ixazomib analogs, boronic ester **14** was then discovered. It contains an eight-membered ring boronic ester pro-drug. In vitro, in silico and in cellulo, it showed low nanomolar activities that were very similar to the two FDA-approved drugs^70^. In vivo assays on TNBC on mice, it revealed great results for tumour necrosis^71^. More recently, Zhou et al. designed a new peptide boronate derivative **15** (Figure 2) from two known active drugs, entinostat (HDAC inhibitor) and bortezomib targeting dual activities. They substituted the zinc-binding moiety of HDAC with the solvent-exposed group of bortezomib^72^ and exhibited excellent proteasome inhibition of IC_50_ values of 1.1 compared to 19.4 nmol/L for bortezomib. Furthermore, compound **15** showed antiproliferative activity against cell lines RPMI-8226, KM3 and U266 in MM with IC_50_ values of 6.66, 10.1 and 4.31 nmol/L, respectively. Interestingly, it showed a remarkable potent antiproliferative activity towards MM cell line KM3/BTZ with an IC_50_ value of 8.98 nmol/L compared to 226 nmol/L for bortezomib. This two-inhibitors combination design revealed a promising candidate for the treatment of MM and further improvements are expected in the near future.

Wang et al. reported recently the synthesis of a series of dipeptide boronic acid esters and investigated their ability to inhibit the β5 subunit of 20S proteasome as potential oral proteasome inhibitors for the treatment of MM^73^. A new prodrug was found to be stable enough in simulated gastric juice and simulated intestinal juice and displayed strong anti-RPMI-8226 activity both *in vitro* and in vivo in comparison to the known ixazomib citrate. This new compound also showed good pharmacokinetic properties, with an oral bioavailability of 34% and elimination half-lives suitable for development as an oral drug. In vivo mouse xenograft models of RPMI-8226 showed that compound 18 u effectively inhibited tumour growth without obvious side effects, making it a potentially effective oral prodrug for MM treatment.

Llona-Minguez et al. used HTS-adapted Malachite Green assay and subsequent optimisation steps discovered a unique boronic acid derivative **16** that inhibits dCTPase (IC_50_ 0.057 mmol/L) and enhances cellular efficacy with EC_50_ of 0.046 mmol/L and kills HL60 leukaemia cells^74^. Although the results were promising, it showed moderate solubility in water (52 mmol/L) and low stability (11% remaining after 4 h) in plasma. In this regard, the boronic acid group was protected with *N*-methyliminodiacetic acid (MIDA) to form the MIDA-boronate ester **17**, which enhanced the aqueous solubility and plasma stability to >100 µmol/L and 86% respectively without affecting the inhibitory activity of dCTPase (IC_50_ = 0.047 mmol/L).

In addition, Zhang et al. designed a new benzoxaborole derivative **18** (Figure 2) using SAR studies and found a remarkable antiproliferation activity towards ovarian cancer cells with an IC_50_ value of 21 nmol/L^75^. Further studies showed nearly 200-fold selectivity towards cancer cells, effectively inhibited colony formation and induced cancer cell apoptosis. Compound **18** was also investigated in vivo and provided efficacy and low toxicity for tumour xenograft mouse models with 2 and 10 mg/kg dosages.

A pinacolate boronic acid derivative **19** is a prodrug of Endoxifen, that is designed and reported by Zhang and co-workers as an effective hormone therapy for breast cancer^76^. Endoxifen is a selective oestrogen receptor modulator (SERM) that failed in clinical trials due to its rapid first-pass metabolism via O-glucuronidation, resulting in poor bioavailability. Compound **19** provided a 40-fold increase in endoxifen concentration making it more of an efficient drug candidate for further investigations. An overall clinical and biomedical presentation of all B-containing anticancer drugs and prodrugs is presented in Table 2.

**Table 2.** Health-promoting anticancer activities of the organoboronic acids/esters drugs and prodrugs.

Drugs/prodrugs/ compound no	Commercial/ abbreviated name	Status	Year of approval/ clinical trials	Cancer type/ type of cell lines	Type of medication/dose	Mechanism of action	Reference
Bortezomib/**1**	Velcade®	Approved	FDA in 2003/Health Canada in 2008	Multiple myeloma	Injectable medication/1.3 mg/m²	20S Proteasome inhibitor/CT-L inhibitor	^40^ ^,^ ^41^ ^,^ ^43^ ^,^ ^45^ ^,^ ^62^
Ixazomib/**2**	Ninlaro®	Approved	FDA in 2015/Health Canada in 2016	Multiple myeloma	Oral medication/2.3 mg, 3 mg or 4 mg	20S proteasome inhibitor/CT-L, T-L and C-L inhibitor	^44–46^ ^,^ ^52^ ^,^ ^62^
Talabostat/**11**	PT-100/Val-Boro-Pro	Failed phase III clinical trials	2006	Non-Hodgkins lymphoma, leukaemia, melanoma, lung and pancreatic cancers	Oral medication/300 µg	DPP and FAP inhibitor	^62^ ^,^ ^63^ ^,^ ^77^
Delanzomib/**12**	CEP-18769	Passed phase I but failed phase II clinical trials	2013	Solid tumours, MM, non-Hodgkins lymphoma	Oral medication/1.5 mg	Proteasome and NF-KB α inhibitor	^67^ ^,^ ^78^
**13**	Boronic acid derivative	Currently under pre-clinical trials	NA	lung (cell lines: A549, 95D), colon (cell line: HCT116), leukaemia (cell line: MGC803), gastric (cell line: MGC803, MKN45), hepatoma (cell line: BEL7404), ovarian (cell line: SKOV3), breast (cell line: MDA-MB-231), liver (HepG2), and pancreatic cancer (cell line: SW1990)	Tested over six concentrations based on three experiments (refer to the reference for more details)	Proteasome inhibitor	^69^
**14**	Boronic ester derivative	pre-clinical trials	NA	In vitro, in silico, and in cellulo showed low nanomolar activities similar to bortezomib and ixazomib. In vivo: great results of tumour necrosis on mice assays with TNBC	NA	NA	^70^ ^,^ ^71^
**15**	Peptide boronate derivative	Pre-clinical trials	NA	Multiple myeloma (cell lines: RPMI-8226, U266 and KM3/BTZ)	NA	Proteasome inhibitor/HDAC inhibitor	^72^
**16**	Boronic acid derivative	Pre-clinical trials	NA	Leukaemia	NA	dCTPase inhibitor	^74^
**17**	MIDA-boronate ester	Pre-clinical trials	NA	Leukaemia	NA	dCTPase inhibitor	^74^
**18**	Benzoxaborate derivative	Pre-clinical trials	NA	Ovarian cancer	NA	Proteasome inhibitor	^75^
**19**	Boron pinacolate derivative	Pre-clinical trials	NA	Breast cancer	NA	A prodrug of endoxifen	^76^
Boronphenylalanine/**20**	BPA	Approved	2012	Breast, bone, head, and neck cancers	Injectable medication/200 mg/kg	Upregulate LAT-1 expression	^79–83^
Sodium boroncaptate/**21** boronphenylalanine-fructose/**22**	BSH/BPA-F	Approved	2007	Thyroid, head, and neck cancers, and liver metastases	Injectable medication/BPA-F 900 mg/kg and BSH 100 mg/kg	Upregulate LAT-1 expression	^79–83^
Calcium fructoborate (CF)/**23**	Borate derivative	Pre-clinical trials	NA	Prostate, lung, cervical, and breast cancer (cell line: MDA-MB-231)	IC_50_ = 5 mM	Inhibits superoxide, interleukin-1β, interleukin-6 and nitric oxide release.	^84^ ^,^ ^85^
Boronate belinostat/**24**	Boron pinacolate derivative	Pre-clinical trials	NA	Breast, lung and cervical cancer (cell lines: MDA-MB-231, MCF-7, A549, and HeLa)	IC_50_ =10–50 µM	Inhibits the mentioned cell lines	^75^ ^,^ ^86^
Boronate 5-fluorouracil/**25**	Boronic acid and boron pinacolate derivatives	Pre-clinical trials	NA	Breast cancer (cell lines: MCF-7, MDA-MB-468, OVCAR-3)	IC_50_ =50 µM	Inhibits the mentioned cell lines	^86^
Boronate doxorubicin/**26**	Boron pinacolate derivative	Pre-clinical trials	NA	Cervical cancer (cell line: HeLa), and breast cancer (cell lines: 4T1 MDF-7)	IC_50_ =0.3 µM	Inhibits the mentioned cell lines	^76^ ^,^ ^87^
Boronate diethyldithiocarbamate/**27**	Boron pinacolate derivative	Pre-clinical trials	NA	Breast cancer (cell line: 4T1)	IC_50_ = <100 mM	Inhibits cancer cell line 4T1 in vivo and in vitro	^88^
Boronate chlormethine/**28**	Boronic acid and boron pinacolate derivatives	Pre-clinical trials	NA	Leukaemia (cell line: SR), lung cancer (cell line NCI-H460)	IC_50_ =10 µM	Inhibits the mentioned cell lines	^16^ ^,^ ^18^ ^,^ ^89^
Benzyloxycarbonyl crizotinib/**29**	Boronic acid derivative	Pre-clinical trials	NA	Lung cancer (cell line H2228) and cell line RUMH	IC_50_ =3**–**15 µM	Inhibits the mentioned cell lines	^90^ ^,^ ^91^
Boronated theranostic/**30**	Boron pinacolate derivative	Pre-clinical trials	NA	Cervical cancer (cell line: HeLa)	IC_50_ =30 µM	Inhibits the HeLa cell line	^92^
Decaboranate phenoxyacetanilide/**31**	Decaborane derivative	Pre-clinical trials	NA	Colon and cervical cancer (cell line: HeLa)	IC_50_= 0.74-µM (cervical)/IC_50_= 5 µM (colon)	IC_50_ and GI_50_ inhibition	^93^

*Notes:* FDA: Food and Drug Administration; CT-L: chymotrypsin-like; T-L: trypsin-like; C-L: caspase-like; FAP: fibroblast activation protein-alpha; DPP: dipeptidyl peptidase; MM: multiple myeloma; NF-KB α: nuclear factor kappa B alpha; TNBC: triple-negative breast cancer; MIDA: *N*-Methylimidodiacetic; HDAC: histone deacetylase; dCTPase: deoxycytidine triphosphatase; BPA: boronophenylalanine; LAT-1: large neutral amino acid transporter; GI_50_: half maximal growth inhibitory concentration; IC_50_: half maximal inhibitory concentration.

## Effective boron-containing drug and prodrug candidates in cancer treatments

### Multiple myeloma

Multiple Myeloma (MM, cancer of plasma cells) is a type of bone marrow cancer that may affect different areas of the body like the ribs, skull, pelvis and spine^94^. MM is a malignant hematological B cell type of cancer that is characterised by the proliferation of abnormal antibodies called monoclonal cells and their infiltration of the bone marrow. They are made with no control and do not respond to or fight off infections in the body^95^. They are mostly secreting non-functional clonal immunoglobulins in heavy chains and, in some cases clonally by light chains. There is a rare non-secretarial MM variant^96^. It is the 3rd most common type of hematological cancer after non-Hodgkin’s lymphoma and leukaemia. According to the study by Knauf et al., in Germany, 6000 patients are diagnosed each year and there is a five-year survival rate of 40%^96^. Treatment initiation starts by considering if patients have hypercalcemia, renal insufficiency, anaemia, or bone disease, this is called the CRAB criteria. Patients without any CRAB symptoms are kept under active surveillance^97^^,^^98^.

High-dose chemotherapy with autologous peripheral blood stem transplantation is often the first choice of treatment when it comes to physically fit patients (< 70 years)^95^^,^^97^. Another type of treatment is proteasome inhibitors, they stop the development of proteasome proteins that help in the proliferation of the myeloma cells. Proteasome inhibitors specifically target the myeloma cells to stop replicating, making it less likely to damage healthy cells in the body, unlike chemotherapy^95^. In this regard, bortezomib **1** and ixazomib **2** are the only two FDA-approved boron-containing medications made specifically for the treatment of MM but may also be used for other types of cancer (Figure 2). Other types of treatment include immunomodulatory drugs, CAR T-cell therapy, monoclonal antibodies, HDAC inhibitors, nuclear export inhibitors, and finally drug conjugates, which are the combination of two drugs^72^^,^^98^ MM is still considered an incurable disease because it goes into remission periods with relapses and progression which can lead to disease resistance^66^.

### Breast cancer

Breast cancer, like other malignancies, can enter and thrive in the tissue surrounding the breast. It can also spread to other parts of the body and cause additional tumours to grow. Surgery is often the first line of defence against breast cancer. Chemotherapy, radiation, or in rare situations, hormonal or targeted therapies are generally administered after surgery.

One of the targeted therapies includes BNCT to treat local invasive malignant cancer^99^. BNCT is a type of treatment based on a radiation technique that is employed for different types of cancers including brain and neck cancers. It usually involves placing an organoboron derivative enriched 10B isotope (the non-radioactive boron) into a tumour. Upon irradiation with low-energy (<0.5 eV) thermal neutrons cause the release of α-particles from 10B nuclei^82^. In cases with oligometastatic breast cancer, BNCT may permit the selective elimination of several tumours with a therapeutic purpose. BNCT could also be useful in treating breast cancer-related metastatic brain lesions^83^. Fujimoto et al. reported the therapy of a 65-year-old lady had an MRI taken and showed a loco-regional relapse of previously treated breast cancer in her left axilla. Two months after she completed therapy; it revealed a marked decline in tumour mass as well as a decreased level of pain. Therefore, BNCT is a novel therapy technique that may be useful in the treatment of several breast cancer disease cases^100^. A recent in-vitro study conducted by Kar et al. investigated the potential use of boric acid (BA) as a treatment for glioblastoma multiform (GBM), a malignant brain tumour with a poor prognosis^101^. The study examined the effects of various doses of BA on glioblastoma cells, specifically its impact on cytotoxicity, ferroptosis, apoptosis, and semaphorin-neuropilin signalling pathways. The results showed that BA triggered ferroptosis in glioblastoma cells in a dose-dependent manner, leading to reduced proliferation and increased apoptosis. These findings suggest that boron compounds have a dual role in the neurotoxicity and anti-tumour effects on glioblastoma cells, and may hold potential as a treatment option for glioblastoma.

**Figure 3.**
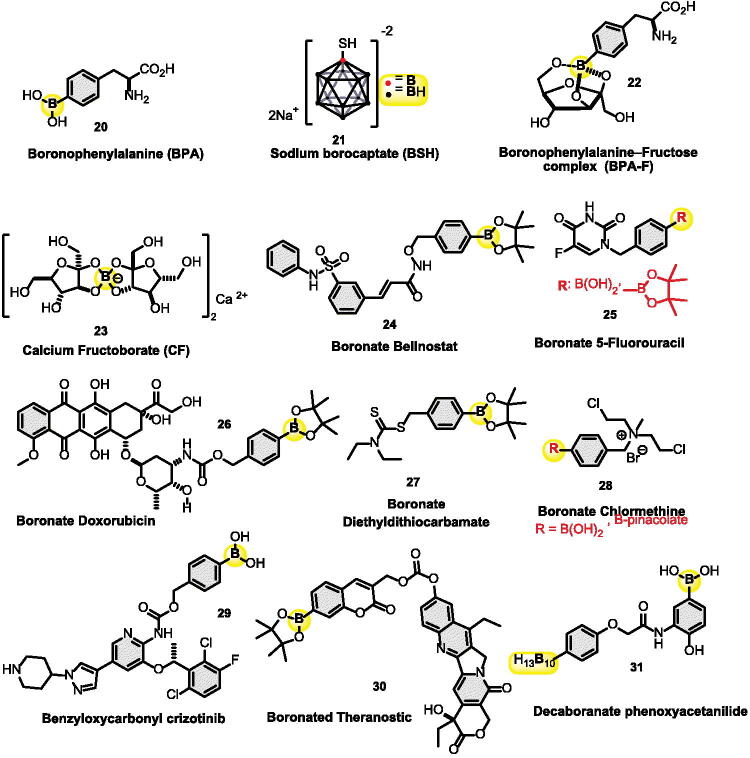
Organoboron derivatives in breast, lung, cervical, and colon cancer treatments.

There have been many boron delivery agents tested in BNCT, however, two compounds Boronophenylalanine **20** (BPA, Figure 3) and sodium borocaptate **21** (BSH), showed great results in cancer cell death and are currently in clinical trials^80^. Hosmane and co-workers developed another perspective direction using carborane-appended 5-thio-D-glucopyranose (5-TDGP) derivatives^102^. A biological screening of these derivatives with human hepatocellular carcinoma cells (SK-Hep1) revealed that 5-TDGP is a better boron carrier than standard D-glucopyranose, making it a promising future BNCT agent.

In vivo injections of BPA are made with BPA-fructose complex **22** (BPA-F, Figure 3) for IV administration in BNCT^81^. Fructose is given with BPA because it has great water solubility^79^. It gets localised to target and uptake cancer cells with the help of L-amino acid transport (LAT-1). The BFA-F **22** has been examined in clinical trials in patients with thyroid cancer, head and neck cancer, and liver metastases^82^. Although no clinical trials have been conducted so far employing BPA as a boron carrier in breast cancer, preclinical research examining LAT-1 expression has shown that it is upregulated in malignant breast cancer tissues when compared to neighbouring normal tissue analogues. BPA-F absorption and cellular boron accumulation were observed to counterpart LAT-1 expression in a bone metastasis model of breast cancer to engage xenografted breast cancer cell lines in mice tibia. In this animal study, peak intracellular boron concentrations were measured 1 h after IV injection of BPA-F and resulted in the tumour growth to significantly decrease in the trial group^103^. However, it is not currently approved for use in humans, and research on its side effects is limited. Some potential side effects of BPA-F in animal studies have been reported such as nausea and vomiting, as well as changes in blood chemistry and hematological parameters. BPA-F has also been shown to cross the blood-brain barrier and accumulate in brain tissue, which could have implications for its potential use in BNCT for brain tumours^104^.

Boron in the diet has also been shown to prevent breast cancer via regulating the sex hormones, on top of that, taking 10 mg/day of boron is recommended^105^. The hormone oestrogen plays an important role in increasing the proliferation of normal and neoplastic breast cells. Natural oestrogen, called 17 β-oestradiol, is responsible for the development of the breasts during puberty, it has been shown that increased levels of 17 β-oestradiol increase the risk of women getting diagnosed with breast cancer^106^^,^^107^. Other forms of boron related to breast cancer include, calcium fructoborate (CF) **23**, shown in Figure 3. It is a natural product from plants that is made as a nutritional supplement or made through chemical synthesis. CF is used as anticancer agent in breast, prostate, lung, and cervical cancer. It works by inhibiting MDA-MB-231 cell line in breast cancer (Inhibits superoxide, interleukin-1 β, interleukin-6 and nitric oxide release). CF enters the cells by a sugar transporter and when inside the cell, it becomes an antioxidant and overexpresses apoptotic proteins to kill the cancer cells^84^^,^^85^. It is generally considered safe for human consumption. In one clinical study, participants took CF supplements for 90 days, and no significant adverse effects were reported. In another study, participants took high doses of CF (up to 28 mg/kg body weight) for 14 days, and again, no significant adverse effects were reported^84^^,^^108^.

Boronate belinostat **24** and boronate 5-fluorouracil **25** derivatives have been both shown to inhibit growth in mice breast cancer cell lines^86^^,^^109^. Boronate belinostat **24** prodrug in vivo was found to be more potent than belinostat alone, it inhibits and reduces the tumour volume in the MCF-7 xenograft tumour model. This prodrug targets MDA-MB-231 and MCF-7 breast cancer cell lines as well as A549 lung cancer cell line, and HeLa for cervical cancer cell line^109^. Some potential side effects of belinostat may include fatigue, nausea, vomiting, diarrhoea, loss of appetite, and hematological toxicities such as anaemia, thrombocytopenia, or neutropenia. In rare cases, severe hypersensitivity reactions or pulmonary toxicity may occur^109^.

Boronate 5-fluorouracil **25** (5-FU) was also found to inhibit OVCAR-3 cancer cell lines in mice ovarian cancer^106^. Some potential side effects of 5-FU may include gastrointestinal disturbances such as nausea, vomiting, diarrhoea, or constipation. 5-FU can also cause myelosuppression, which may result in a decrease in white blood cells, red blood cells, or platelets. Other potential side effects may include skin reactions, neurotoxicity, cardiotoxicity, or infusion reactions^110^.

Furthermore, another prodrug called boronate doxorubicin **26**, is made by masking the medication doxorubicin with a boronate moiety since it was shown to reduce doxorubicin toxicity and improve its stability. Moreover, it was shown to inhibit 4T1 and MDF-7 breast cancer cell lines at a half maximal inhibitory concentration (IC_50_) = 0.3 µM, 30 times higher than doxorubicin alone^87^. Boronate doxorubicin has been investigated in preclinical studies, but there is limited data on its safety and efficacy in humans. In preclinical, some potential side effects of boronate doxorubicin may include myelosuppression, which may result in a decrease in white blood cells, red blood cells, or platelets. Other potential side effects may include gastrointestinal disturbances such as nausea, vomiting, diarrhoea, or constipation^87^.

Recently, Pan et al. investigated the inhibition of boronate diethyldithiocarbamate (BDDC) derivative **27** and found that it also targets the 4T1 breast cancer cells. In **27**, boronic pinacolate is being used as a carrier in the prodrug, with the help of hydrogen peroxide, it gets oxidised by the existing H_2_O_2_ in the cancer cell to release diethyldithiocarbamate (DTC). DTC chelates with the available Cu(II) in the cell to give the active anticancer drug Cu(DTC)2. Remarkably, it showed specific toxicity towards cancer cells with low toxicity to normal organs. The Cu(DTC)2 combined with chemotherapy and oxidative stress amplification, showed to be successful in cancer inhibition and apoptosis both in vivo and *in vitro*^88^.

### Prostate cancer

Prostate cancer is cancer of the prostate gland. It is classified into two main types: benign prostatic hyperplasia (BPH) and prostate cancer. BPH is considered the most benign condition in the prostate, and it has been related to the action of the enzyme 5-reductase on testosterone, leading to the production of dihydroxy testosterone (DHT), which is 10 times more potent than testosterone and enhances the proliferation of stromal and glandular elements. On the other hand, prostate cancer progresses from an early, androgen-dependent, organ-confined disease to a highly invasive, androgen-independent, metastatic disease. The metastasising of tumour cells is the major cause of cancer-related deaths^111^.

Cui and co-workers reported that the high concentration of boron in males reduces the probability of prostate cancer by 54% compared to males with low boron concentrations in the body^112^. The advancement of primary prostate cancer is reliant on the androgen receptor (AR) and prostate-specific membrane antigen (PSMA). The decreased expression of AR and PSMA inhibits prostate cell proliferation.

In preliminary investigations, it has been discovered that bortezomib suppresses 26S proteasome activity and serves as a down regulator for both PSMA and AR^113^. In a dose-dependent manner, bortezomib reduced the development of two prostate cancer cell lines. AR and PSMA play critical roles in allowing cells to tolerate anticancer medications like docetaxel and bortezomib. When detecting apoptotic cells using fluorescence-activated cell sorting, the apoptosis-inducing effect on treated cells with bortezomib alone was more potent than that observed with docetaxel alone. According to these findings, bortezomib triggered death in cells expressing AR and PSMA by reducing protein expression in both. It seems that, to effectively treat cancer, bortezomib should be used with other anticancer medications. It has also reportedly shown to make human prostate cancer cells more sensitive to the effects of radiation when used in combination with docetaxel^113^.

Another form is boron nitride spheres, they seem to have promising chemoprevention reagent that causes a decrease in cell viability and enhances prostate cancer cell apoptosis^114^. Another form of boron is boric acid, it has shown a slowdown of prostate tumours in mice by significantly inhibiting the degradation of fibronectin^13^^,^^115^. The same authors showed that expression of insulin-like growth factor 1 (IGF-1) in tumours was markedly reduced by boric acid. Upon exposure to both low-and high-dose boron supplementation, prostate-specific antigen (PSA) levels dropped by an average of 87%, while tumour size declined by an average of 31.5%^116^.

McAuley et al. found that phenylboronic acid is a more potent inhibitor than boric acid in targeting metastatic and proliferative properties of prostate cancer cells^117^. It decreases the activities of RhoA, Rac1 and Cdc42 in DU-145 metastatic prostate cancer cell lines, but not in normal RWPE −1 prostate cells.

Kimberly et al. reported another dietary boron intake study and found that ER Ca^+2^ depletion occurred after the treatment of DU-145 prostate cancer cells with the physiological concentrations of boric acid, and authors suggested a plausible mode of action that boric acid identifies the eIF2α/ATF pathway^118^. Lastly, Barranco et al. looked at animal and cell-line studies and found that boron inhibited the proliferation of prostate cancer cell lines DU-145 (an androgen-independent line) and LNCaP (an androgen-dependent cell line)^119^. Additionally, peptide boronic acid derivatives can inhibit prostate cancer by hindering the PSA^120^.

### Lung cancer

Lung cancer (LC) is the number one leading cause of cancer death worldwide with about 20% mortality rate and an 18.6% five-year survival rate. It is classified into two different types, small-cell lung cancer (SCLC) and non-small cell lung cancer (NSCLC). SCLC is more aggressive and has only a 5% survival rate over five years.^121^. It is calculated that ∼71% of cancer-related deaths belong to low and middle-developed countries^121^ where many of the treatments options are available for patients such as surgery, chemotherapy, radiotherapy, and drug therapy, however, the cancer mortality rate is extremely high^122^ and accordingly, no effective treatment can be used to date for the treatment of lung cancer patients, especially for those diagnosed with the SCLC type. State-of-the-art discoveries of new targeted drugs and smart drug candidates’ use are still limited due to their high cost and unwanted side effects^123^. Therefore, LC remains one of the top deadly cancers with the highest mortality rate where development of new drug candidates as new therapeutic treatments is crucial.

The use of boron intake has an inverse relation with lung cancer in women. Although the mechanism is still unclear, it has a role in regulating the levels of oestradiol and testosterone hormone levels in women^124^. Boronate chlormethine **28** (Figure 3) was synthesised as a nitrogen mustard prodrug by Kuang and co-workers^89^. It was found to inhibit cancer cell lines at 10 μM concentrations. It inhibited 90% of SR cancer cell lines in leukaemia and 85% NCI-H460 of cancer cell lines in NSCLC. The mechanism of action is investigated and found that **28** get activated first through the high level of reactive oxygen species (ROS) located in the cancer cell or by hydrogen peroxide (H_2_O_2_). The boron group gets hydrolysed and leads to form aziridinium ring in a few steps, which is linked to forming alkylating DNA^16^^,^^18^^,^^89^. Boronate chlormethine **28** has been investigated in preclinical studies, but there is limited data on its safety and efficacy in humans. Some potential side effects of boronate chlormethine may include myelosuppression, which may result in a decrease in white blood cells, red blood cells, or platelets. Other potential side effects may include gastrointestinal disturbances such as nausea, vomiting, diarrhoea, or constipation, as well as skin reactions^16^^,^^18^^,^^89^.

Recently, Bielec et al. modified crizotinib, an anticancer drug marketed under the name Xalkori® for the treatment of NSCLC, to enhance the cell cancer selectivity and to reduce the unwanted side effects. The authors modified crizotinib by forming a carbamate group with amine, giving benzyloxycarbonyl crizotinib **29** as shown in Figure 3. The boronic acid group got activated first by hydrogen peroxide within 1h of incubation and through the same mechanism. This prodrug has an inhibition (IC_50_) of cancer cell lines H2228 and RUMH between 3–15 µM^90^^,^^91^. Additionally, Cebeci et al. recently reported an *in vitro* study on the effect of boric acid (BA), sodium pentaborate pentahydrate (NaB), and sodium perborate tetrahydrate (SPT) against SCLC cell line using DMS-114 cells^125^. Authors found that boric acid, NaB, and SPT cause apoptosis in SCLC cell lines through the upregulation of pro-apoptotic genes Bax and Casp-3 and downregulation of the anti-apoptotic genes BIRC2, BIRC5, and Bcl-2. TP63 gene expression is also notably upregulated and that mediates the p53-dependent apoptosis. Finally, both BA and NaB cause cell cycle arrest in the G2/M phase and SPT in the Sub-G1 phase. It is worth noting that this study showed great potential for SCLC treatment without affecting the healthy cells.

### Cervical and colon cancers

Cervical cancer is the fourth most common cancer in women. It is strongly linked to infection with high-risk of human papillomaviruses (HPV). It is estimated that 570 000 women were diagnosed with this cancer; 311 000 died from the disease. Although the mortality is high, it is considered one of the most curable types of cancer. Several boron-containing prodrugs were reported and showed promising results in cervical cancer therapy. For instance, boronate doxorubicin **26** (Figure 3) was reported to target HeLa cancer cell lines in cervical cancer after boron got activated through H_2_O_2_^71^. Additionally, boronated theranostic prodrug **30** is also reported to target Hela cancer cell lines. It can cure 100% of cervical cancer^91^^,^^92^. Some potential side effects may include gastrointestinal disturbances such as nausea, vomiting, diarrhoea, or constipation, as well as skin reactions. Colon and prostate cancers are connected^126^^,^^127^. In addition, long-term androgen deprivation therapy for prostate cancer is linked to an increased risk of colorectal cancer^126^.

Decaboranate phenoxyacetanilide **31** is one of the prodrugs that has been synthesised and tested against several cancer cell lines. The cell viability assay showed that **31** inhibits Hela cancer cell lines in cervical cancer with IC_50_ 0.74 µM and colon cancer with growth inhibitory (GI_50_ = 5.0 µM)^93^. Interestingly, boric acid shows the ability to inhibit several kinds of cancer cell lines for colon cancer. Besides, boric acid inhibits 46.6% of the Caco-2 cancer cell line at concentrations of 1.0 mM after seven days^128^. Furthermore, boric acid has IC_50_ at a concentration of 50 mM showed that after two days, it inhibited CCL-233 human colon cancer cells^128^. Finally, boric acid inhibits SW-480 human colon cancer cells at a concentration of 75 mM with an inhibition rate of 45.5%^129^.

## Conclusion

This article provides an overview of the challenges and opportunities of using boron-containing medications in enzyme inhibition-based drug discovery and highlights the importance of enzyme inhibition in cancer therapy. It discusses the different types of enzyme inhibitors, including proteasome, chymotrypsin-like, caspase-like, dipeptidyl peptidase, nuclear factor kappa B alpha, histone deacetylase, deoxycytidine triphosphatase, fibroblast activation protein-alpha, large neutral amino acid transporter, trypsin-like and tyrosine kinases their mechanisms of action, as well as the strategies for drug designing for therapeutic use.

The results of published articles are encouraging enough to envisage the future of clinical research in cancer treatment using organoboronic acid/ester drugs and prodrugs. Boronic acid/ester compounds are usually unionised under physiological pH and therefore, they can be utilised for chemotherapy and chemoprevention. It has been long ignored by medicinal chemists due to their toxicity concerns in drug design and discovery. The toxicity study by Brigham et al.^130^ revealed that boric acid has a lethal dose (LD) of 2660 mg/kg (rats, oral), which is comparable to the LD of table salt (3000 mg/kg rats, oral).

Other challenges can be faced during the drug development of boronic acid drugs and prodrugs. The challenges are due to boronic acid being unstable, highly reactive, and having a short shelf life. They are highly electrophilic and react with various nucleophiles, and therefore could be degraded before binding to the target. This non-specific reactivity limited their selectivity at the active site of target enzymes. Boronic acids are relatively difficult to synthesise especially aliphatic ones. To solve this synthetic issue, installing the boronic acid group to the drug core structure is usually made at the last step during the synthesis. This is due to its liability under different reaction conditions. As a result, it must be protected and stabilised by making boronic esters and boron heterocycles such as benzoboroxoles. However, some protected boronic acid (boronic ester) prodrugs are remarkably stable, therefore, under physiological conditions, it is hard to hydrolyse them and make the free boronic acid derivative, which results in a slow-release element in a medicinal pathway. Since the FDA approved bortezomib (Velcade®) medication in 2003, increased research activities have been accomplished on the design of aliphatic boronic acid/ester derived from amino-acid and peptide derivatives or even incorporating the boronic acid group into their drug design approaches seeking more reactivity.

One possible mechanism that describes the remarkable activity of boron-containing prodrugs against cancer cells is the enzymatic inhibition through reversable electrophilicity. Nonetheless, research working on developing new boron-containing medications for cancer must consider the factors highlighted in this review. These investigations will confidently shed more light on the advantages of boron in drug scaffold and inspire pharmaceutical and medicinal allies. With the enormous scope of B-containing derivatives and the role of the boronic acid group in obtaining new candidates for treating cancer, the hope arises with boron drug candidates’ inhibition mechanisms to have a great impact on cancer in the near future. In summary, boron drugs and prodrugs can target a variety of enzymes and have shown promise as potential cancer therapeutics.

## References

[1] Watanabe T, Momose I. [Boronic acid as a promising class of chemical entity for development of clinical medicine for targeted therapy of cancer]. Yakugaku Zasshi. 2022;142(2):145–153. 35110451 10.1248/yakushi.21-00173-3

[2] (a) Sun X, Peng Y, Zhao J, Xie Z, Lei X, Tang G. Discovery and development of tumor glycolysis rate-limiting enzyme inhibitors. Bioorg Chem. 2021;112:104891. 33940446 10.1016/j.bioorg.2021.104891

[3] (a) Schwarzel WC, Kruggel WG, Brodie HJ. Studies on the mechanism of estrogen biosynthesis. 8. The development of inhibitors of the enzyme system in human placenta. Endocrinology. 1973;92(3):866–880. 4267111 10.1210/endo-92-3-866

[4] (a) Lacal PM, Graziani G. Therapeutic implication of vascular endothelial growth factor receptor-1 (VEGFR-1) targeting in cancer cells and tumor microenvironment by competitive and non-competitive inhibitors. Pharmacol Res. 2018;136:97–107. 30170190 10.1016/j.phrs.2018.08.023

[5] McClure JJ, Li X, Chou CJ. Advances and challenges of HDAC inhibitors in cancer therapeutics. Adv Cancer Res. 2018;138:183–211. 29551127 10.1016/bs.acr.2018.02.006

[6] Zhang L, Zhang J, Jiang Q, Zhang L, Song W. Zinc binding groups for histone deacetylase inhibitors. J Enzyme Inhib Med Chem. 2018;33(1):714–721. 29616828 10.1080/14756366.2017.1417274 PMC6009916

[7] Hunt CD. Dietary boron: progress in establishing essential roles in human physiology. J Trace Elem Med Biol. 2012;26(2-3):157–160. 22658717 10.1016/j.jtemb.2012.03.014

[8] Pizzorno L. Nothing boring about boron. Integr Med. 2015;14(4):35–48. PMC4712861 26770156

[9] Ying X, Cheng S, Wang W, Lin Z, Chen Q, Zhang W, Kou D, Shen Y, Cheng X, Rompis FA, et al. Effect of boron on osteogenic differentiation of human bone marrow stromal cells. Biol Trace Elem Res. 2011;144(1-3):306–315. 21625915 10.1007/s12011-011-9094-x

[10] (a) Hall DG. Structure, properties, and preparation of boronic acid derivatives. In: Hall DG, editor. Boronic acids. Wiley‐VCH Verlag GmbH & Co. KGaA; 2011. p. 1–133.

[11] Fernandes GFS, Denny WA, Dos Santos JL. Boron in drug design: recent advances in the development of new therapeutic agents. Eur J Med Chem. 2019;179:791–804. 31288128 10.1016/j.ejmech.2019.06.092

[12] Tanaka K. The proteasome: overview of structure and functions. Proc Jpn Acad Ser B Phys Biol Sci. 2009;85(1):12–36. 10.2183/pjab.85.12 PMC3524306 19145068

[13] Gonzalez A, Peters U, Lampe JW, White E. Boron intake and prostate cancer risk. Cancer Causes Control. 2007;18(10):1131–1140. 17851770 10.1007/s10552-007-9052-2

[14] Scorei RI, Popa R. Jr. Boron-containing compounds as preventive and chemotherapeutic agents for cancer. Anticancer Agents Med Chem. 2010;10(4):346–351. 19912103 10.2174/187152010791162289

[15] (a) António JPM, Russo R, Carvalho CP, Cal P, Gois PMP. Boronic acids as building blocks for the construction of therapeutically useful bioconjugates. Chem Soc Rev. 2019;48(13):3513–3536. 31157810 10.1039/c9cs00184k

[16] Peiró Cadahía J, Previtali V, Troelsen NS, Clausen MH. Prodrug strategies for targeted therapy triggered by reactive oxygen species. Medchemcomm. 2019;10(9):1531–1549. 31673314 10.1039/c9md00169g PMC6786010

[17] Andina D, Leroux JC, Luciani P. Ratiometric fluorescent probes for the detection of reactive oxygen species. Chemistry. 2017;23(55):13549–13573. 28561437 10.1002/chem.201702458

[18] Peng X, Gandhi V. ROS-activated anticancer prodrugs: a new strategy for tumor-specific damage. Ther Deliv. 2012;3(7):823–833. 22900465 10.4155/tde.12.61 PMC3566582

[19] Cao S, Christiansen R, Peng X. Substituent effects on oxidation-induced formation of quinone methides from arylboronic ester precursors. Chemistry. 2013;19(27):9050–9058. 23670793 10.1002/chem.201300539

[20] Zielonka J, Sikora A, Hardy M, Joseph J, Dranka BP, Kalyanaraman B. Boronate probes as diagnostic tools for real time monitoring of peroxynitrite and hydroperoxides. Chem Res Toxicol. 2012;25(9):1793–1799. 22731669 10.1021/tx300164j PMC3501381

[21] (a) Sikora A, Zielonka J, Lopez M, Joseph J, Kalyanaraman B. Direct oxidation of boronates by peroxynitrite: mechanism and implications in fluorescence imaging of peroxynitrite. Free Radic Biol Med. 2009;47(10):1401–1407. 19686842 10.1016/j.freeradbiomed.2009.08.006 PMC3375817

[22] Dong C, Zhou Q, Xiang J, Liu F, Zhou Z, Shen Y. Self-assembly of oxidation-responsive polyethylene glycol-paclitaxel prodrug for cancer chemotherapy. J Control Release. 2020;321:529–539. 32109513 10.1016/j.jconrel.2020.02.038

[23] Hanna RD, Naro Y, Deiters A, Floreancig PE. Alcohol, aldehyde, and ketone liberation and intracellular cargo release through peroxide-mediated α-boryl ether fragmentation. J Am Chem Soc. 2016;138(40):13353–13360. 27636404 10.1021/jacs.6b07890 PMC7075644

[24] (a) Mosey RA, Floreancig PE. Versatile approach to α-alkoxy carbamate synthesis and stimulus-responsive alcohol release. Org Biomol Chem. 2012;10(39):7980–7985. 22936329 10.1039/c2ob26571k PMC3532511

[25] Efremenko Y, Mirsky VM. 3-Thienylboronic acid as a receptor for diol-containing compounds: a study by isothermal titration calorimetry. Chemosensors. 2022;10(7):251.

[26] (a) Liu J, Zheng S, Akerstrom VL, Yuan C, Ma Y, Zhong Q, Zhang C, Zhang Q, Guo S, Ma P, et al. Fulvestrant-3 boronic acid (ZB716): an orally bioavailable selective estrogen receptor downregulator (SERD). J Med Chem. 2016;59(17):8134–8140. 27529700 10.1021/acs.jmedchem.6b00753 PMC5499704

[27] (a) Alouane A, Labruère R, Le Saux T, Schmidt F, Jullien L. Self-immolative spacers: kinetic aspects, structure-property relationships, and applications. Angew Chem Int Ed Engl. 2015;54(26):7492–7509. 26053475 10.1002/anie.201500088

[28] (a) Baker SJ, Ding CZ, Akama T, Zhang YK, Hernandez V, Xia Y. Therapeutic potential of boron-containing compounds. Future Med Chem. 2009;1(7):1275–1288. 21426103 10.4155/fmc.09.71

[29] (a) Brown HC, Malhotra SV, Ramachandran PV. Organoboranes for synthesis 17. Generality of hydroboration-amination for the conversion of terpenes into enantiomerically pure terpenylamines. Their utility for gas chromatographic analysis of chiral carboxylic acids. Tetrahedron Asymmetry. 1996;7(12):3527–3534.

[30] (a) Al-Zoubi RM, Al-Jammal WK, McDonald R. Regioselective synthesis of ortho-iodobiphenylboronic acid derivatives: a superior catalyst for carboxylic acid activation. New J Chem. 2020;44(9):3612–3623.

[31] Baldock C, de Boer GJ, Rafferty JB, Stuitje AR, Rice DW. Mechanism of action of diazaborines. Biochem Pharmacol. 1998;55(10):1541–1549. 9633989 10.1016/s0006-2952(97)00684-9

[32] Jabbour A, Steinberg D, Dembitsky VM, Moussaieff A, Zaks B, Srebnik M. Synthesis and evaluation of oxazaborolidines for antibacterial activity against *Streptococcus mutans*. J Med Chem. 2004;47(10):2409–2410. 15115381 10.1021/jm049899b

[33] Benkovic SJ, Baker SJ, Alley MRK, Woo Y-H, Zhang Y-K, Akama T, Mao W, Baboval J, Rajagopalan PTR, Wall M, et al. Identification of borinic esters as inhibitors of bacterial cell growth and bacterial methyltransferases, CcrM and MenH. J Med Chem. 2005;48(23):7468–7476. 16279806 10.1021/jm050676a

[34] Baker SJ, Zhang YK, Akama T, Lau A, Zhou H, Hernandez V, Mao W, Alley MR, Sanders V, Plattner JJ. Discovery of a new boron-containing antifungal agent, 5-fluoro-1,3-dihydro-1-hydroxy-2,1- benzoxaborole (AN2690), for the potential treatment of onychomycosis. J Med Chem. 2006;49(15):4447–4450. 16854048 10.1021/jm0603724

[35] Al-Zoubi RM, Al-Zoubi MS, Jaradat KT, McDonald R. Design, synthesis and X-ray crystal structure of iodinated benzoboroxole derivatives by consecutive metal–iodine exchange of 3,4,5-triiodoanisole. Eur J Org Chem. 2017;2017(38):5800–5808.

[36] Akama T, Baker SJ, Zhang YK, Hernandez V, Zhou H, Sanders V, Freund Y, Kimura R, Maples KR, Plattner JJ. Discovery and structure-activity study of a novel benzoxaborole anti-inflammatory agent (AN2728) for the potential topical treatment of psoriasis and atopic dermatitis. Bioorg Med Chem Lett. 2009;19(8):2129–2132. 19303290 10.1016/j.bmcl.2009.03.007

[37] Dogan EE. Computational bioactivity analysis and bioisosteric investigation of the approved breast cancer drugs proposed new design drug compounds: increased bioactivity coming with silicon and boron. Lett Drug Des Discov. 2021;18(6):551–561.

[38] Ferlay J, Colombet M, Soerjomataram I, Mathers C, Parkin DM, Piñeros M, Znaor A, Bray F. Estimating the global cancer incidence and mortality in 2018: GLOBOCAN sources and methods. Int J Cancer. 2019;144(8):1941–1953. 30350310 10.1002/ijc.31937

[39] Kuczynski EA, Sargent DJ, Grothey A, Kerbel RS. Drug rechallenge and treatment beyond progression–implications for drug resistance. Nat Rev Clin Oncol. 2013;10(10):571–587. 23999218 10.1038/nrclinonc.2013.158 PMC4540602

[40] U.S. Food and Drug Administration. Drug approval package. Maryland: FDA; 2003.

[41] Ortho Biotech - Division of Janssen-Ortho Inc. VELCADE for multiple myeloma receives front-line approval in Canada. Canada: Johnson and Johnson; 2008.

[42] (a) Adams J. The development of proteasome inhibitors as anticancer drugs. Cancer Cell. 2004;5(5):417–421. 15144949 10.1016/s1535-6108(04)00120-5

[43] Chen D, Frezza M, Schmitt S, Kanwar J, Dou QP. Bortezomib as the first proteasome inhibitor anticancer drug: current status and future perspectives. Curr Cancer Drug Targets. 2011;11(3):239–253. 21247388 10.2174/156800911794519752 PMC3306611

[44] Williams C, Luis M, Lopez I, Coca A. The chemistry of organoboron species: classification and basic properties. In: Gandelman M, Marek I, editors. PATAI’s chemistry of functional groups. Delaware: John Wiley & Sons; 2021. p. 1–21.

[45] Bross PF, Kane R, Farrell AT, Abraham S, Benson K, Brower ME, Bradley S, Gobburu JV, Goheer A, Lee SL, et al. Approval summary for bortezomib for injection in the treatment of multiple myeloma. Clin Cancer Res. 2004;10(12 Pt 1):3954–3964. 15217925 10.1158/1078-0432.CCR-03-0781

[46] Shirley M. Ixazomib: first global approval. Drugs. 2016;76(3):405–411. 26846321 10.1007/s40265-016-0548-5

[47] Takeda Pharmaceutical Company Ltd. Health Canada approves Ninlaro (ixazomib) for use in relapsed/refractory multiple myeloma. Ontario (CA): International Myeloma Foundation; 2016.

[48] Muz B, Ghazarian RN, Ou M, Luderer MJ, Kusdono HD, Azab AK. Spotlight on ixazomib: Potential in the treatment of multiple myeloma (Review). Drug Des Devel Ther. 2016;10:217–226. 10.2147/DDDT.S93602 PMC4714737 26811670

[49] Kupperman E, Lee EC, Cao Y, Bannerman B, Fitzgerald M, Berger A, Yu J, Yang Y, Hales P, Bruzzese F, et al. Evaluation of the proteasome inhibitor MLN9708 in preclinical models of human cancer. Cancer Res. 2010;70(5):1970–1980. 20160034 10.1158/0008-5472.CAN-09-2766

[50] Dick LR, Fleming PE. Building on bortezomib: second-generation proteasome inhibitors as anti-cancer therapy. Drug Discov Today. 2010;15(5-6):243–249. 20116451 10.1016/j.drudis.2010.01.008

[51] (a) Dimopoulos MA, Grosicki S, Jędrzejczak WW, Nahi H, Gruber A, Hansson M, Gupta N, Byrne C, Labotka R, Teng Z, et al. All-oral ixazomib, cyclophosphamide, and dexamethasone for transplant-ineligible patients with newly diagnosed multiple myeloma. Eur J Cancer. 2019;106:89–98. 30471652 10.1016/j.ejca.2018.09.011

[52] Gupta N, Yang H, Hanley MJ, Zhang S, Liu R, Kumar S, Richardson PG, Skacel T, Venkatakrishnan K. Dose and schedule selection of the oral proteasome inhibitor ixazomib in relapsed/refractory multiple myeloma: clinical and model-based analyses. Target Oncol. 2017;12(5):643–654. 28803351 10.1007/s11523-017-0524-3 PMC5610674

[53] Ludwig H, Avet-Loiseau H, Bladé J, Boccadoro M, Cavenagh J, Cavo M, Davies F, de la Rubia J, Delimpasi S, Dimopoulos M, et al. European perspective on multiple myeloma treatment strategies: update following recent congresses. Oncologist. 2012;17(5):592–606. 22573721 10.1634/theoncologist.2011-0391 PMC3360899

[54] US Food and Drug Administration. FDA drug approval process infographic. USA: Food and Drug Administration; 2014.

[55] Hughes JP, Rees S, Kalindjian SB, Philpott KL. Principles of early drug discovery. Br J Pharmacol. 2011;162(6):1239–1249. 21091654 10.1111/j.1476-5381.2010.01127.x PMC3058157

[56] Bhat V, Chatterjee J. The use of in silico tools for the toxicity prediction of potential inhibitors of SARS-CoV-2. Altern Lab Anim. 2021;49(1-2):22–32. 33845649 10.1177/02611929211008196 PMC8047515

[57] Cengiz Seval G, Beksac M. The safety of bortezomib for the treatment of multiple myeloma. Expert Opin Drug Saf. 2018;17(9):953–962. 30118610 10.1080/14740338.2018.1513487

[58] VELCADE. Prescribing information. Ontario (CA): Takeda Pharmaceuticals America; 2022.

[59] BORTEZOMIB FOR INJECTION. Bortezomib for Injection. Toronto (CA): Teva Canada Limited; 2006.

[60] Jouni H, Aubry MC, Lacy MQ, Vincent Rajkumar S, Kumar SK, Frye RL, Herrmann J. Ixazomib cardiotoxicity: a possible class effect of proteasome inhibitors. Am J Hematol. 2017;92(2):220–221. 27859518 10.1002/ajh.24608

[61] (a) Nguyen MN, Nayernama A, Jones SC, Kanapuru B, Gormley N, Waldron PE. Proteasome inhibitor-associated thrombotic microangiopathy: a review of cases reported to the FDA adverse event reporting system and published in the literature. Am J Hematol. 2020;95(9):E218–E222. 32291777 10.1002/ajh.25832

[62] Plescia J, Moitessier N. Design and discovery of boronic acid drugs (Review). Eur J Med Chem. 2020;195:112270. 32302879 10.1016/j.ejmech.2020.112270

[63] Cunningham CC. Talabostat. Expert Opin Investig Drugs. 2007;16(9):1459–1465. 10.1517/13543784.16.9.1459 17714031

[64] Flentke GR, Munoz E, Huber BT, Plaut AG, Kettner CA, Bachovchin WW. Inhibition of dipeptidyl aminopeptidase IV (DP-IV) by Xaa-boroPro dipeptides and use of these inhibitors to examine the role of DP-IV in T-cell function. Proc Natl Acad Sci U S A. 1991;88(4):1556–1559. 1671716 10.1073/pnas.88.4.1556 PMC51058

[65] Rashmi R, Kumar S, Karunagaran D. Ectopic expression of Bcl-XL or Ku70 protects human colon cancer cells (SW480) against curcumin-induced apoptosis while their down-regulation potentiates it. Carcinogenesis. 2004;25(10):1867–1877. 15205359 10.1093/carcin/bgh213

[66] Offidani M, Corvatta L, Morè S, Olivieri A. Novel experimental drugs for treatment of multiple myeloma (Review). J Exp Pharmacol. 2021;13:245–264. 33727866 10.2147/JEP.S265288 PMC7955760

[67] Vogl DT, Martin TG, Vij R, Hari P, Mikhael JR, Siegel D, Wu KL, Delforge M, Gasparetto C. Phase I/II study of the novel proteasome inhibitor delanzomib (CEP-18770) for relapsed and refractory multiple myeloma. Leuk Lymphoma. 2017;58(8):1872–1879. 28140719 10.1080/10428194.2016.1263842

[68] (a) Hatakeyama S, Ohyama C, Minagawa S, Inoue T, Kakinuma H, Kyan A, Arai Y, Suga T, Nakayama J, Kato T, et al. Functional correlation of trophinin expression with the malignancy of testicular germ cell tumor. Cancer Res. 2004;64(12):4257–4262. 15205339 10.1158/0008-5472.CAN-04-0732

[69] Han LQ, Yuan X, Wu XY, Li RD, Xu B, Cheng Q, Liu ZM, Zhou TY, An HY, Wang X, et al. Urea-containing peptide boronic acids as potent proteasome inhibitors. Eur J Med Chem. 2017;125:925–939. 27769033 10.1016/j.ejmech.2016.10.023

[70] (a) Bani-Yaseen AD. The supramolecular host-guest complexation of Vemurafenib with β-cyclodextrin and cucurbit[7]uril as drug photoprotecting systems: a DFT/TD-DFT study. Comput Theor Chem. 2020;1191:113026.

[71] Lei M, Feng H, Bai E, Zhou H, Wang J, Qin Y, Zhang H, Wang X, Liu Z, Hai O, et al. Discovery of a novel dipeptidyl boronic acid proteasome inhibitor for the treatment of multiple myeloma and triple-negative breast cancer. Org Biomol Chem. 2019;17(3):683–691. 30601533 10.1039/c8ob02668h

[72] Zhou Y, Liu X, Xue J, Liu L, Liang T, Li W, Yang X, Hou X, Fang H. Discovery of peptide boronate derivatives as histone deacetylase and proteasome dual inhibitors for overcoming bortezomib resistance of multiple myeloma. J Med Chem. 2020;63(9):4701–4715. 32267687 10.1021/acs.jmedchem.9b02161

[73] Wang X, Zhang W, Wen T, Miao H, Hu W, Liu H, Lei M, Zhu Y. Design and discovery of novel dipeptide boronic acid ester proteasome inhibitors, an oral slowly-released prodrug for the treatment of multiple myeloma. Eur J Med Chem. 2023;250:115187. 36806958 10.1016/j.ejmech.2023.115187

[74] Llona-Minguez S, Höglund A, Jacques SA, Johansson L, Calderón-Montaño JM, Claesson M, Loseva O, Valerie NCK, Lundbäck T, Piedrafita J, et al. Discovery of the first potent and selective inhibitors of human dCTP pyrophosphatase 1. J Med Chem. 2016;59(3):1140–1148. 26771665 10.1021/acs.jmedchem.5b01741 PMC4753678

[75] Zhang J, Zhang J, Hao G, Xin W, Yang F, Zhu M, Zhou H. Design, synthesis, and structure-activity relationship of 7-propanamide benzoxaboroles as potent anticancer agents. J Med Chem. 2019;62(14):6765–6784. 31264855 10.1021/acs.jmedchem.9b00736

[76] Zhang C, Zhong Q, Zhang Q, Zheng S, Miele L, Wang G. Boronic prodrug of endoxifen as an effective hormone therapy for breast cancer. Breast Cancer Res Treat. 2015;152(2):283–291. 26071758 10.1007/s10549-015-3461-9 PMC4524496

[77] Point Therapeutics. Study of talabostat + docetaxel versus docetaxel in stage IIIB/IV non-small cell lung cancer (NSCLC) after failure of platinum-based chemotherapy. Indianapolis (Indiana): NIH, ClinicalTrials.gov; 2007.

[78] Vogl DT, Martin TG, Vij R, Hari P, Mikhael JR, Siegel D, Wu KL, Delforge M, Gasparetto C. Phase I/II study of the novel proteasome inhibitor delanzomib (CEP-18770) for relapsed and refractory multiple myeloma. Leuk Lymphoma. 2017;58(8):1872–1879. 10.1080/10428194.2016.1263842 28140719

[79] Mori Y, Suzuki A, Yoshino K, Kakihana H. Complex formation of p-boronophenylalanine with some monosaccharides. Pigment Cell Res. 1989;2(4):273–277. 2508079 10.1111/j.1600-0749.1989.tb00203.x

[80] Nedunchezhian K, Aswath N, Thiruppathy M, Thirugnanamurthy S. Boron neutron capture therapy - a literature review. J Clin Diagn Res. 2016;10(12):Ze01–Ze04. 10.7860/JCDR/2016/19890.9024 PMC5296588 28209015

[81] Shimosegawa E, Isohashi K, Naka S, Horitsugi G, Hatazawa J. Assessment of (10)B concentration in boron neutron capture therapy: potential of image-guided therapy using (18)FBPA PET. Ann Nucl Med. 2016;30(10):749–755. 27586407 10.1007/s12149-016-1121-8

[82] Wongthai P, Hagiwara K, Miyoshi Y, Wiriyasermkul P, Wei L, Ohgaki R, Kato I, Hamase K, Nagamori S, Kanai Y. Boronophenylalanine, a boron delivery agent for boron neutron capture therapy, is transported by ATB0,+, LAT1 and LAT2. Cancer Sci. 2015;106(3):279–286. 25580517 10.1111/cas.12602 PMC4376436

[83] Malouff TD, Seneviratne DS, Ebner DK, Stross WC, Waddle MR, Trifiletti DM, Krishnan S. Boron neutron capture therapy: a review of clinical applications (Review). Front Oncol. 2021;11:1–11. 10.3389/fonc.2021.601820 PMC7952987 33718149

[84] Scorei IR. Calcium fructoborate: plant-based dietary boron as potential medicine for cancer therapy. Front Biosci. 2011;3(1):205–215. 10.2741/s145 21196370

[85] Scorei RI, Rotaru P. Calcium fructoborate–potential anti-inflammatory agent. Biol Trace Elem Res. 2011;143(3):1223–1238. 21274653 10.1007/s12011-011-8972-6

[86] Ai Y, Obianom ON, Kuser M, Li Y, Shu Y, Xue F. Enhanced tumor selectivity of 5-fluorouracil using a reactive oxygen species-activated prodrug approach. ACS Med Chem Lett. 2019;10(1):127–131. 30655959 10.1021/acsmedchemlett.8b00539 PMC6331168

[87] Ye M, Han Y, Tang J, Piao Y, Liu X, Zhou Z, Gao J, Rao J, Shen Y. A tumor-specific cascade amplification drug release nanoparticle for overcoming multidrug resistance in cancers. Adv Mater. 2017;29(38):1702342. 10.1002/adma.201702342 28833669

[88] Pan Q, Zhang B, Peng X, Wan S, Luo K, Gao W, Pu Y, He B. A dithiocarbamate-based H2O2-responsive prodrug for combinational chemotherapy and oxidative stress amplification therapy. Chem Commun. 2019;55(92):13896–13899. 10.1039/c9cc05438c 31675022

[89] Kuang Y, Balakrishnan K, Gandhi V, Peng X. Hydrogen peroxide inducible DNA cross-linking agents: targeted anticancer prodrugs. J Am Chem Soc. 2011;133(48):19278–19281. 22035519 10.1021/ja2073824 PMC3265938

[90] Bielec B, Poetsch I, Ahmed E, Heffeter P, Keppler BK, Kowol CR. Reactive oxygen species (ROS)-sensitive prodrugs of the tyrosine kinase inhibitor crizotinib. Molecules. 2020;25(5):1149. 32143435 10.3390/molecules25051149 PMC7179202

[91] Maslah H, Skarbek C, Pethe S, Labruère R. Anticancer boron-containing prodrugs responsive to oxidative stress from the tumor microenvironment. Eur J Med Chem. 2020;207:112670. 32858470 10.1016/j.ejmech.2020.112670

[92] Kim EJ, Bhuniya S, Lee H, Kim HM, Cheong C, Maiti S, Hong KS, Kim JS. An activatable prodrug for the treatment of metastatic tumors. J Am Chem Soc. 2014;136(39):13888–13894. 25238144 10.1021/ja5077684

[93] Shimizu K, Maruyama M, Yasui Y, Minegishi H, Ban HS, Nakamura H. Boron-containing phenoxyacetanilide derivatives as hypoxia-inducible factor (HIF)-1alpha inhibitors. Bioorg Med Chem Lett. 2010;20(4):1453–1456. 20083404 10.1016/j.bmcl.2009.12.037

[94] National Health Service (NHS). Multiple myeloma. England: NHS; 2021.

[95] National Comprehensive Cancer Network (NCCN). NCCN guidelines: multiple myeloma. Pennsylvania (USA): NCCN; 2013.

[96] Knauf W, Abenhardt W, Aldaoud A, Nusch A, Grugel R, Münz M, Hartmann H, Marschner N, TLN Study Group. Treatment of non-transplant patients with multiple myeloma: routine treatment by office-based haematologists in Germany–data from the prospective Tumour Registry Lymphatic Neoplasms (TLN). Oncol Res Treat. 2014;37(11):635–636, 638–644. 25486127 10.1159/000368315

[97] (a) Kortüm MEH, Naumann R, Peest D, Liebisch P, Goldschmidt H. DGHO-leitlinie multiples myelom. Washington (DC): Blood, American Society of Hematology; 2010.

[98] Harousseau JL, Dreyling M, ESMO Guidelines Working Group. Multiple myeloma: ESMO Clinical Practice Guidelines for diagnosis, treatment and follow-up. Ann Oncol. 2010;21 Suppl 5:v155–v157. 20555068 10.1093/annonc/mdq178

[99] Nielsen FH. Update on human health effects of boron (Review). J Trace Elem Med Biol. 2014;28(4):383–387. 25063690 10.1016/j.jtemb.2014.06.023

[100] Fujimoto T, Maekawa Y, Takao S, Hori S, Andoh T, Sakurai Y, Tanaka H, Kinashi Y, Masunaga S, Ichikawa H, et al. Anti-tumor effect of boron neutron capture therapy (BNCT) on axillary lymph node metastasis of breast cancer. KURRI Prog Rep. 2016;44:53.

[101] (a) Hacioglu C, Kar F, Kar E, Kara Y, Kanbak G. Effects of curcumin and boric acid against neurodegenerative damage induced by amyloid beta (1-42). Biol Trace Elem Res. 2021;199(10):3793–3800. 33237490 10.1007/s12011-020-02511-2

[102] Satapathy R, Dash BP, Bode BP, Byczynski EA, Hosmane SN, Bux S, Hosmane NS. New classes of carborane-appended 5-thio-D-glucopyranose derivatives. Dalton Trans. 2012;41(29):8982–8988. 22722329 10.1039/c2dt30874f

[103] Andoh T, Fujimoto T, Satani R, Suzuki M, Wada K, Sudo T, Sakurai Y, Tanaka H, Takata T, Ichikawa H. Preclinical study of boron neutron capture therapy for bone metastasis using human breast cancer cell lines. Appl Radiat Isot. 2020;165:109257. 32777739 10.1016/j.apradiso.2020.109257

[104] (a) Barth RF, Coderre JA, Vicente MG, Blue TE. Boron neutron capture therapy of cancer: current status and future prospects. Clin Cancer Res. 2005;11(11):3987–4002. 15930333 10.1158/1078-0432.CCR-05-0035

[105] (a) Khaliq H, Juming Z, Ke-Mei P. The physiological role of boron on health (Review). Biol Trace Elem Res. 2018;186(1):31–51. 29546541 10.1007/s12011-018-1284-3

[106] Fernandez SV, Russo J. Estrogen and xenoestrogens in breast cancer (Review). Toxicol Pathol. 2010;38(1):110–122. 19933552 10.1177/0192623309354108 PMC2907875

[107] Russo J, Russo IH. The role of estrogen in the initiation of breast cancer. J Steroid Biochem Mol Biol. 2006;102(1-5):89–96. 17113977 10.1016/j.jsbmb.2006.09.004 PMC1832080

[108] (a) Scorei R, Ciubar R, Ciofrangeanu CM, Mitran V, Cimpean A, Iordachescu D. Comparative effects of boric acid and calcium fructoborate on breast cancer cells. Biol Trace Elem Res. 2008;122(3):197–205. 18176783 10.1007/s12011-007-8081-8

[109] Zhang C, Guo S, Zhong Q, Zhang Q, Hossain A, Zheng S, Wang G. Metabolism and pharmacokinetic study of the boron-containing prodrug of belinostat (Zl277), a pan hdac inhibitor with enhanced bioavailability. Pharmaceuticals. 2019;12(4):180. 31817969 10.3390/ph12040180 PMC6958523

[110] (a) Feng Y, Zhang Y, Zhou D, Chen G, Li N. MicroRNAs, intestinal inflammatory and tumor. Bioorg Med Chem Lett. 2019;29(16):2051–2058. 31213403 10.1016/j.bmcl.2019.06.013

[111] Karan D, Lin MF, Johansson SL, Batra SK. Current status of the molecular genetics of human prostatic adenocarcinomas (Short Survey). Int J Cancer. 2003;103(3):285–293. 10.1002/ijc.10813 12471610

[112] Cui Y, Winton MI, Zhang ZF, Rainey C, Marshall J, De Kernion JB, Eckhert CD. Dietary boron intake and prostate cancer risk. Oncol Rep. 2004;11(4):887–892. 15010890

[113] Kuroda K, Liu H. The proteasome inhibitor, bortezomib, induces prostate cancer cell death by suppressing the expression of prostate-specific membrane antigen, as well as androgen receptor. Int J Oncol. 2019;54(4):1357–1366. 30720063 10.3892/ijo.2019.4706

[114] (a) Li X, Wang X, Zhang J, Hanagata N, Wang X, Weng Q, Ito A, Bando Y, Golberg D. Hollow boron nitride nanospheres as boron reservoir for prostate cancer treatment. Nat Commun. 2017;8(1):13936–13947. 28059072 10.1038/ncomms13936 PMC5228389

[115] Ozel AB, Dagsuyu E, Aydın PK, Bugan I, Bulan OK, Yanardag R, Yarat A. Brain boron level, DNA content, and myeloperoxidase activity of metformin-treated rats in diabetes and prostate cancer model. Biol Trace Elem Res. 2022;200(3):1164–1170. 33860456 10.1007/s12011-021-02708-z

[116] Gallardo-Williams MT, Chapin RE, King PE, Moser GJ, Goldsworthy TL, Morrison JP, Maronpot RR. Boron supplementation inhibits the growth and local expression of IGF-1 in human prostate adenocarcinoma (LNCaP) tumors in nude mice. Toxicol Pathol. 2004;32(1):73–78. 14713551 10.1080/01926230490260899

[117] McAuley EM, Bradke TA, Plopper GE. Phenylboronic acid is a more potent inhibitor than boric acid of key signaling networks involved in cancer cell migration. Cell Adh Migr. 2011;5(5):382–386. 21975546 10.4161/cam.5.5.18162 PMC3218604

[118] Henderson KA, Kobylewski SE, Yamada KE, Eckhert CD. Boric acid induces cytoplasmic stress granule formation, eIF2α phosphorylation, and ATF4 in prostate DU-145 cells. Biometals. 2015;28(1):133–141. 25425213 10.1007/s10534-014-9809-5 PMC4300416

[119] Barranco WT, Eckhert CD. Boric acid inhibits human prostate cancer cell proliferation. Cancer Lett. 2004;216(1):21–29. 15500945 10.1016/j.canlet.2004.06.001

[120] LeBeau AM, Singh P, Isaacs JT, Denmeade SR. Potent and selective peptidyl boronic acid inhibitors of the serine protease prostate-specific antigen. Chem Biol. 2008;15(7):665–674. 18635003 10.1016/j.chembiol.2008.05.020 PMC3360951

[121] Sung H, Ferlay J, Siegel RL, Laversanne M, Soerjomataram I, Jemal A, Bray F. Global cancer statistics 2020: GLOBOCAN estimates of incidence and mortality worldwide for 36 cancers in 185 countries. CA Cancer J Clin. 2021;71(3):209–249. 33538338 10.3322/caac.21660

[122] Suda K, Mitsudomi T. Successes and limitations of targeted cancer therapy in lung cancer. Prog Tumor Res. 2014;41:62–77. 24727987 10.1159/000355902

[123] (a) Devarakonda S, Rotolo F, Tsao M-S, Lanc I, Brambilla E, Masood A, Olaussen KA, Fulton R, Sakashita S, McLeer-Florin A, et al. Tumor mutation burden as a biomarker in resected non-small-cell lung cancer. J Clin Oncol. 2018;36(30):2995–3006. 30106638 10.1200/JCO.2018.78.1963 PMC6804865

[124] (a) Mahabir S, Spitz MR, Barrera SL, Dong YQ, Eastham C, Forman MR. Dietary boron and hormone replacement therapy as risk factors for lung cancer in women. Am J Epidemiol. 2008;167(9):1070–1080. 18343880 10.1093/aje/kwn021 PMC3390773

[125] Cebeci E, Yüksel B, Şahin F. Anti-cancer effect of boron derivatives on small-cell lung cancer. J Trace Elem Med Biol. 2022;70:126923. 35007916 10.1016/j.jtemb.2022.126923

[126] Moot AR, Polglase A, Giles GG, Garson OM, Thursfield V, Gunter D. Men with colorectal cancer are predisposed to prostate cancer. ANZ J Surg. 2003;73(5):289–293. 12752284 10.1046/j.1445-2197.2003.t01-1-02621.x

[127] Gillessen S, Templeton A, Marra G, Kuo YF, Valtorta E, Shahinian VB. Risk of colorectal cancer in men on long-term androgen deprivation therapy for prostate cancer. J Natl Cancer Inst. 2010;102(23):1760–1770. 21068432 10.1093/jnci/djq419 PMC2994861

[128] Çiğel A, Bilgin MD, Ek RO. Evaluation of the anti-cancer and biological effects of boric acid on colon cancer cell line. Meandros. 2020;21(3):238–243.

[129] Sevimli M, Bayram D, Özgöçmen M, Armağan I, Semerci Sevimli T. Boric acid suppresses cell proliferation by TNF signaling pathway mediated apoptosis in SW-480 human colon cancer line. J Trace Elem Med Biol. 2022;71:126958. 35219976 10.1016/j.jtemb.2022.126958

[130] Eula Bingham, editor. B. C. E. Patty’s toxicology. Toxicology 2012;6(6):895–896.

